# A golden opportunity: Corporate sustainability reporting as a key lever to address nature-related risks

**DOI:** 10.1007/s13280-026-02376-0

**Published:** 2026-03-14

**Authors:** Beatrice Crona, Stephen Polasky, Giorgio Parlato, Véronique Blum, Maxime Mathon

**Affiliations:** 1 Stockholm Resilience Centre, Stockholm University, Albanovägen 28, 114 19 Stockholm, Sweden; 2 Global Economic Dynamics and the Biosphere, Royal Swedish Academy of Sciences, Stockholm, Sweden; 3 Department of Applied Economics, University of Minnesota, 231 Ruttan Hall, 1994 Buford Avenue, St. Paul, MN 55108 USA; 4 Centre de Préparation à l’Expertise Comptable, Université Grenoble des Alpes, 2 Place Doyen Gosse, 38000 Grenoble, France; 5 Ascend, 28 Place de la Bourse, 75002 Paris, France

**Keywords:** Corporate sustainability reporting, Disclosures, Environmental social and governance (ESG), Materiality, Reform, Risks

## Abstract

Corporate sustainability reporting currently fails to provide the information needed for companies, their investors and society to address mounting environmental risks because predominant reporting requirements focus heavily on firm-level risks, neglecting cumulative impacts on climate and nature. Recently, sustainability reporting has also been critiqued for the administrative burden it places on companies. Corporate reporting may seem far removed from sustainability science but is, in fact, a critical lever in directing corporate practice. A golden opportunity therefore exists for sustainability science to simultaneously help reduce, refine and improve sustainability reporting by identifying the most prioritized mandatory environmental disclosures and support the development of an open-access disclosure repository. This would radically improve the ability of investors, regulators, and public agencies to appraise environmental pressures, improve corporate accountability, and mitigate growing systemic risks. Treating environmental data with the rigor granted to financial data is fundamental to align capital with sustainability objectives.

**Supplementary Information:**

The online version contains supplementary material available at https://doi.org/10.1007/s13280-026-02376-0.

## Introduction

While the debate over corporate sustainability reporting standards in the EU and elsewhere may seem technical at first glance, its consequences are far-reaching and urgent. Investments steer economic activity, determining what activities grow and shrink. Economic activities in turn drive climate change, loss of biodiversity, and other global environmental change, which create a new risk landscape that exposes companies, the financial system, and societies globally to novel amounts and types of risk (Crona et al. 2021; Almeida et al. 2022; Ranger et al. 2023). Emergence of such risks is evident already today—whether it is Californian wildfires (Wang et al. 2021; Iglesias et al. 2025), increasing frequency and intensity of European hail storms that damage crops and property (Rana et al. 2022; Kopp et al. 2023), or droughts and heatwaves that reap human lives and disrupt transport and production (United Nations Convention to Combat Desertification 2022). For private capital to help change this trend, investors need information that can guide capital allocation toward sustainable practices and ambitious targets (Kılkış et al. 2025) and away from activities contributing to systemic risks.

Today, investors use Environmental, Social, and Governance (ESG) data for multiple purposes, including to align investments with sustainability objectives by informing active ownership, to screen investments and tilt portfolios, or otherwise inform decision-making and the fulfillment of fiduciary responsibilities (CFA Institute 2022; Blum and Mathon 2023). Most ESG information is provided by a small number of ESG rating agencies and data providers in the form of ratings, indices, etc. (Escrig-Olmedo et al. 2019). While investors use such ESG outputs, industry consultations show that they generally consider the underlying corporate data the most valuable information provided by ESG raters (European Commission 2022). This is because the transparency and reliability of ESG data outputs is poor, with significant methodological biases and high divergence between ESG ratings (Chatterji et al. 2016; Berg et al. 2022; European Commission 2022; Crona and Sundström 2023). Raw data in the form of corporate sustainability disclosures allows more flexible use for investors and asset managers, to assess sustainability performance but also to identify impacts and opportunities that lie in mitigating environmental harm.

The current ESG data market is therefore considered dysfunctional because the commoditization of underlying ESG data leads to an information asymmetry between investors and companies (European Commission 2022; Blum and Mathon 2023). However, the observed market failure (c.f Akerlof 1970; Mas-Colell et al. 1995; Bebczuk 2003; Roychowdhury et al. 2019) is not just about asymmetrical information, but also about a failure to generate the type and quality of information necessary to conduct reliable analyses of the novel environmental risk landscape (Wassénius et al. 2024; Kashyap et al. 2025).

The world is transgressing a number of planetary boundaries that threaten to destabilize the environmental conditions within which human societies have evolved and thrived (Rockström et al. 2009; Richardson et al. 2023) Transparent, publicly available, and science-based corporate sustainability disclosures that capture companies’ contributions to cumulative environmental harm, or their nature-positive contributions, are therefore fundamental for companies and investors to take stock of impacts, but also opportunities to address them, and to address both failures described above.

Despite the noted frustration with ESG data markets, ESG investments have risen to significant levels (Bloomberg LP 2025). Even so, the significant inflows of capital into ‘green’ investments have not generated any noticeable abatement in overall environmental degradation (Mace et al. 2018; Richardson et al. 2023). One reason is that current systems of non-financial environmental disclosures and reporting (from hereon referred to as sustainability disclosures/reporting) and their use in ESG measures create major barriers for sustainable investing to deliver on its ambition. Below, we describe four such barriers and propose an alternative way forward.

### Non-financial disclosures are largely voluntary

Prior to the EU adopting the Corporate Sustainability Reporting Disclosures (CSRD), mandatory corporate disclosures were limited to financial and compliance data. A company’s sustainability disclosures, which forms a big part of ESG metrics, are instead reported in annual sustainability reports where companies have been free to decide what information to include (Crona and Sundström 2023). Only recently have reporting frameworks and standards for sustainability information been given wider attention. This includes the European Sustainability Reporting Standards (ESRS), the market-driven Taskforce on Nature-related Financial Disclosures (TNFD), the recently developed sustainability node of the International Financial Reporting Standards called the International Sustainability Standards Board (ISSB), as well as the Global Reporting Initiative (GRI). GRI is a notable exception, as they have developed reporting standards for various social and environmental disclosures since 2000. The European Sustainability Reporting Standards also stand out as they are the only currently mandatory sustainability disclosures in the world.

All sustainability frameworks and standards, including the mandatory ESRS, currently adopt a comply or explain approach. This allows companies to refrain from providing sustainability information, as long as they explain why they do not believe the omitted information is important (financially material) for their organization. An important point here is that while such non-disclosing companies may appear as sustainability laggards by not disclosing various information about their environmental impact (and could plausibly incur some reputational risk if they are a consumer facing brand), the capacity for actually sanctioning such reporting sluggishness currently rests solely on the market. That is, sanctioning is entirely up to investors, many who have a relatively limited understanding of the environmental impacts of various sectors, and who also have limited experience and training in interpreting non-financial information (Schumacher 2020).

### ESG ratings use proprietary methods that are non-transparent and non-standardized

ESG ratings emerged as an analytical service to help investors assess company sustainability information (Escrig-Olmedo et al. 2019). Such services were perceived as necessary given the difficulties for investors in interpreting individual companies’ non-standardized and self-reported sustainability information (Eccles et al. 2020). However, the business model for ESG rating services is based on ownership of proprietary data and methods, which precludes transparency and prevents third-party scrutiny or verification (Escrig-Olmedo et al. 2019). Furthermore, the lack of a standardized rating approach has led to large divergences between alternative ESG metrics (Chatterji et al. 2016; Dimson et al. 2020; El-Hage 2021; Berg et al. 2022). This is due to the fact that the underlying data they use are not the same, despite the new UK and European regulation aimed at regulating ESG rating agencies (*Regulation (EU) 2024/3005; Financial Services and Markets Act *2000). As already noted, instead of supporting decision-making, as intended, this reinforces the information asymmetry between companies and investors and presents investors with multiple versions of sustainability assessments of a company, something which would be inconceivable for financial data.

### ESG metrics measure what is material to the financial bottom line of companies but do not capture many key environmental impacts nor their magnitude, thus obscuring increasing systemic risks

Definitions of what ESG is and should include vary, but the predominant view is that ESG is a framework that helps corporate stakeholders (particularly investors) understand how the company is managing risks and opportunities related to environmental, social, and governance criteria (sometimes called ESG factors) (Principles for Responsible Investment 2018). This view prevails among influential organizations that produce ESG ratings and metrics to inform portfolio-level decision-making, such as MSCI, Sustainalytics/Morningstar, S&P Global, ISS and Moody’s and current ESG practice is thus firmly grounded in corporate risk assessment and focused on business sustainability only (Eccles et al. 2020). Environmental impacts that cannot be directly traced to a financial risk to the firm in the short term are unlikely to be considered (Crona and Sundström 2023). One exception is greenhouse gas emissions, which have become financially material to most firms as societal awareness of the anthropogenic drivers of climate change has developed and climate regulations have materialized (Carney 2015; Task Force on Climate-related Financial Disclosures 2017). Emissions of greenhouse gases can be estimated from fossil-fuel use and other company activities and impacts from different greenhouse gases can be estimated using carbon dioxide equivalent (CO_2_e).

**Fig. 1:**
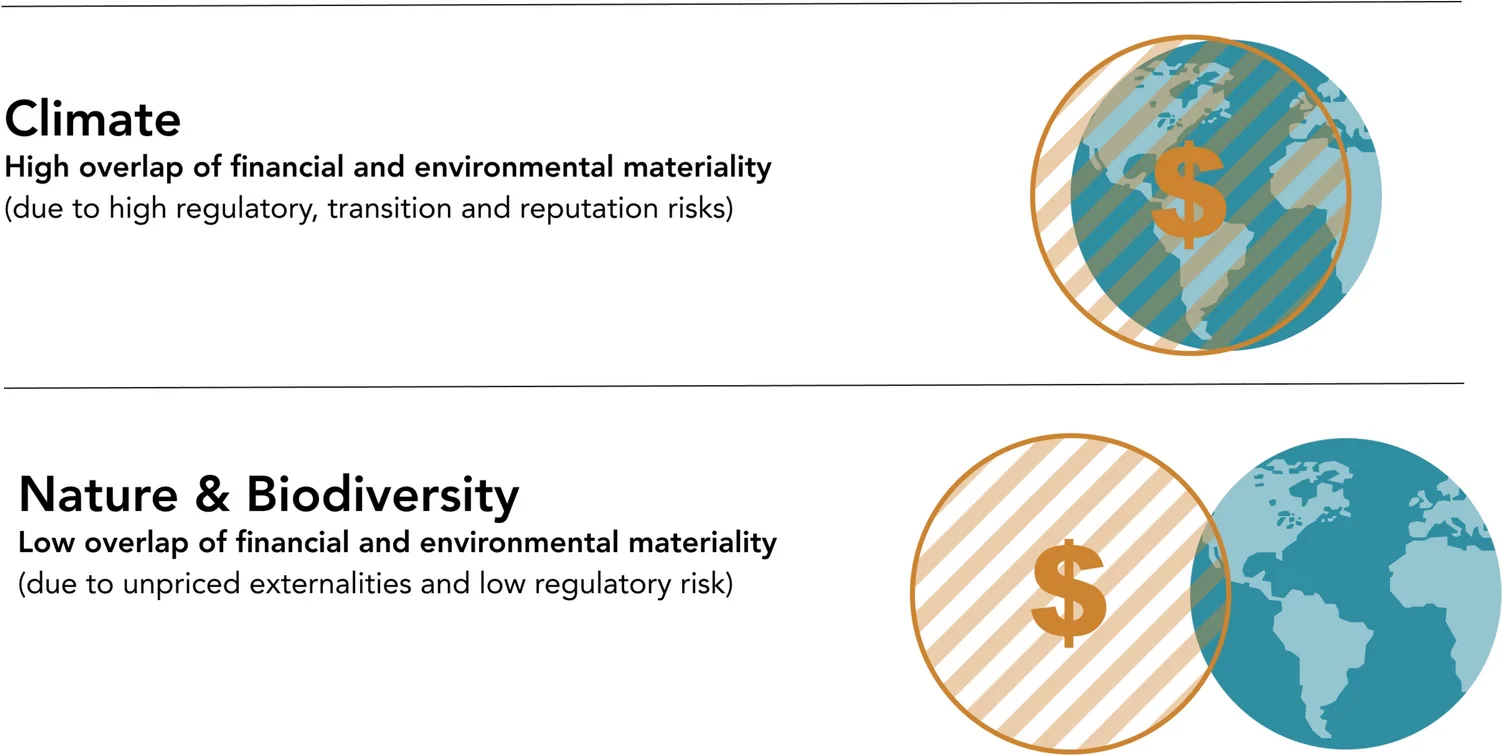
Financial and environmental materiality do not align for most environmental impacts beyond carbon emissions. Environmental materiality refers to information that is essential to assess the status of the environment, regardless of whether it is financially material to the reporting company. While the financial risks of not reporting on greenhouse gas emissions have become so large that financial and environmental materiality largely overlap (top panel), this is not the case for most other environmental pressures and impacts (bottom panel)

In stark contrast to climate change, causal mechanisms linking company activity to other environmental impacts are often more complex and spatially heterogeneous making them less well understood by companies, the general public, and regulatory bodies (Mazor et al. 2018; Dasgupta and Levin 2023). This is evidenced by the fact that many jurisdictions impose limited (or no) regulations on drivers of nature and biodiversity loss, such as habitat change, invasive species, overexploitation, and pollution, resulting in low reputational and regulatory risks to companies (Mazor et al. 2018; Groh et al. 2022; Isbell et al. 2023). Consequently, most environmental impacts remain largely unpriced externalities (Dasgupta and Levin 2023) and do not directly affect the financial bottom line of companies (Fig. 1).

Nature-related impacts arise from economic activities creating pressures on nature through use/appropriation of natural resources (e.g., land, water, biomass), or release of pollutants or substances that alter the functioning of natural systems (e.g., nutrients, chemicals, plastics). These pressures have diverse effects on biodiversity, human health and well-being, and economic productivity (Mazor et al. 2018; IPBES 2019; Groh et al. 2022; Isbell et al. 2023). While each pressure can often be estimated through life-cycle analysis of products or sectors, the individual diverse impacts cannot be represented by a combined single summary impact measure, similar to carbon equivalents.

We refer to disclosures that would provide information that allows for assessment of how companies actually influence environmental conditions as ‘environmentally material information.’ Such environmentally material information could include disclosure by companies of their own measured or estimated environmental pressures known to drive nature change and biodiversity loss (IPBES 2019), and be reported as, e.g., area of land use, volumes of water use, and volumes of released pollutants (see Wassénius et al. 2024). These pressures also relate directly to the transgression of planetary boundaries and have been proposed as the means to translate and operationalize the boundaries into tangible actions by companies (Bai et al. 2024). However, disclosures representing this kind of information are currently not prioritized or widely mandated. This gap between financially and environmentally material information leads to large discrepancies between the actual environmental impacts of a company’s activities and what ESG metrics capture (Fig. 1), presenting a challenge for sustainable investments relying on ESG data.

### Relative measures alone are not sufficient in a world of absolute limits

Another issue with corporate sustainability accounting and ESG measures is the strong focus on relative measures (e.g., carbon intensity measured as GHG emissions/volumes produced or GHG/total revenue) rather than absolute measures of environmental impact (e.g., total carbon emissions) (Bjørn et al. 2017). Relative measures normalize environmental impact by revenue, sales or production volumes and thereby offer investors the advantage of being able to compare the efficiency of resource use across companies. These measures do not, however, divulge a company’s absolute impact (Bjørn et al. 2019). For example, a company with a relatively lower carbon intensity than its sector peers, which reports a year-on-year increase in sales and volumes, might still be increasing its absolute contribution to carbon in the atmosphere. Consequently, relative measures cannot account for the aggregate and cumulative environmental impact of corporate activities (Wassénius et al. 2024). This is highly problematic in a world where science shows clearly that there are finite limits, beyond which anthropogenic pressures are likely to lead to large uncertainties and potentially drastic reductions in the ecosystem functions and services our economies depend on (Rocha et al. 2018; Armstrong McKay et al. 2022). Assessments of total cumulative impacts are also needed to assess our collective progress against globally set targets such as the Global Biodiversity Framework, the Paris Climate Agreement, or planetary boundaries (Richardson et al. 2023), and it is the foundation for reliable risk assessments as called for by the European Central Bank (European Central Bank 2024). Worth mentioning is that any relative measure of impact (pressure) necessitates reporting organizations to have an absolute measure/estimate of pressure. Therefore, disclosing the raw data necessary for calculating a relative measure (e.g., the total water use (numerator) and the revenue or sales volume (denominator)) is the more transparent way of reporting and should be favored, and in line with the call by investors to have raw data for more flexible and bespoke analyses (European Commission 2022).

Recently, market-based initiatives (e.g., Taskforce on Nature-related Financial Disclosures) and regulatory frameworks (ESRS) have encouraged the disclosure of information about drivers of impacts on nature. However, disclosures remain largely voluntary or subject to materiality assessments where individual companies can decide what appears to be financially material and relevant environmental information to report. This leaves such disclosures partial, incomplete, and unreliable for both impact and risk assessments. For example, harmful activities that appear small or represent small revenue streams for any particular company are typically not reported, even though in aggregate, and cumulatively over time, they push society past critical environmental limits (Richardson et al. 2023).

## An institutional landscape in flux

Because of the potentially unprecedented increase in systemic risks from climate change and other global environmental changes, both public and private sector actors have begun to recognize the pressing need for better information about business impacts on people and the environment. This realization was behind the ambition of the European Commission when it launched the European Green Deal in 2019, and later the hitherto unparalleled regulatory ambition of the European Corporate Sustainability Reporting Directive (CSRD), and the closely related Sustainable Finance Disclosure Regulation (SFDR). It also spurred similar developments in the UK, including the 2022 Climate-related Financial Disclosure Regulations (SI 2022 No. 31), the forthcoming Sustainable Disclosure Requirements (SDR) and Green Taxonomy of the UK (UK Government 2024). Despite this realization, the aforementioned corporate reporting standards and frameworks remain company-centric in their reporting, perpetuating the financial materiality focus even for non-financial information, such as environmental and social impacts.

Since their launch, the European Green Deal-related regulations have been critiqued by business associations and conservative political groups for imposing excessive administrative and financial burdens without providing sufficient flexibility or clarity (BusinessEurope 2024). Concerns have been raised that detailed sustainability reporting could stifle competitiveness and innovation (Draghi 2024). In response, the EU 'Omnibus simplification package' was recently released with the aim of refining the CSRD and reducing the reporting burden for companies (BusinessEurope 2024). Proposed simplification measures will now reduce the initial scope of the legislation to include only companies with more than 1000 employees, making sustainability reporting voluntary for all other companies within the union (European Commission 2025). This will vastly reduce the information (in the form of company reported data) that analysts of environmental impact and risk have access to. As a result, it will significantly undermine the possibility of investors investing in EU companies to accurately assess the environmental impact and thus sustainability of their portfolio companies and consequently undermines their ability to accurately assess the risks that these same companies are both contributing to and exposed to.

## Mandatory, scientifically informed environmental disclosures offer unprecedented opportunities

While the slow-down in regulatory development and implementation of corporate sustainability reporting is arguably concerning, it also represents a golden opportunity to take stock, reflect, and improve the system of sustainability reporting. Next, we elaborate a path that can simultaneously balance reduced disclosure volume with the need for high-quality information that supports both environmental needs, economic demands, and broader social goals.

More accurate stock-taking of cumulative absolute impacts of business activity is essential to inform more reliable nature-related risk assessments for company management, investors, regulators, and society at large. Such information would also highlight important areas for urgent corporate action and changed practices. Improved disclosures would thus increase the potential for market discipline by providing investors with clear information about a company’s impacts and its contribution to increases in systemic risks, something that current measures focused on firm-level risks do not allow. By imposing structured reporting requirements, mandatory disclosures act as a catalyst for internal control improvements by enforcing accountability, aligning governance practices, and reducing risks of misreporting (Alruwaili et al. 2023; Wu et al. 2025). As such, they largely determine what is material for a company to include in their internal control and strategy and are therefore key to incentivizing inclusion of social and environmental impact information in target-setting and performance evaluation (i.e., what gets measured gets managed) (Wu et al. 2025).

A system that can generate reliable assessments of both financial and non-financial environmental (and arguably social) impacts, opportunities and risks requires that: (1) companies disclose information that has been scientifically identified as material for assessing environmental status and impacts, (2) non-financial disclosures be mandatory for companies across supply chains, and (3) information is transparently disclosed and publicly available in an open repository. Below, we elaborate on each of these requirements, drawing on environmental and accounting literature. While we recognize that regulatory environments and political discourse in some regions currently make the move toward such a system challenging in the short term, we argue that the articulation of such a vision as an ambitious goal to navigate toward is critical, but currently lacking. Failure to embrace these three criteria will largely undermine the intent of standardized reporting to address the market failure linked to information asymmetry. It will also undercut the ability to enact market discipline and to address the climate and nature-related risks (European Central Bank 2024).

### Environmentally material information based on scientific assessment

Recent findings show that science-based impact disclosures need to account for three key aspects of company activities, which can be simply translated as: *where, what, and how much* (Wassénius et al. 2024). ‘*Where’* refers to information about the location of company activities which is essential because contrary to climate impacts, impacts on nature vary by location.*’What’* refers to information about the kinds of activities that occur at each location, since the environmental impacts of activities within, e.g., agriculture, mining, manufacturing, and service sectors differ in terms of their environmental impact per unit of production. ‘*How much’* refers to information about the scale of the operation as this, along with information about where and what, determines total (absolute) impact (Bjørn et al. 2017). Current frameworks and standards (e.g., TNFD, GRI, ESRS) do recognize a need for information about the location of company assets, but location-specific information is only demanded if impacts are considered financially material by the reporting company. Furthermore, without the additional information about *what* and *how much*, location information alone is not sufficient to assess the scale and nature of a company’s environmental impacts.

**Fig. 2:**
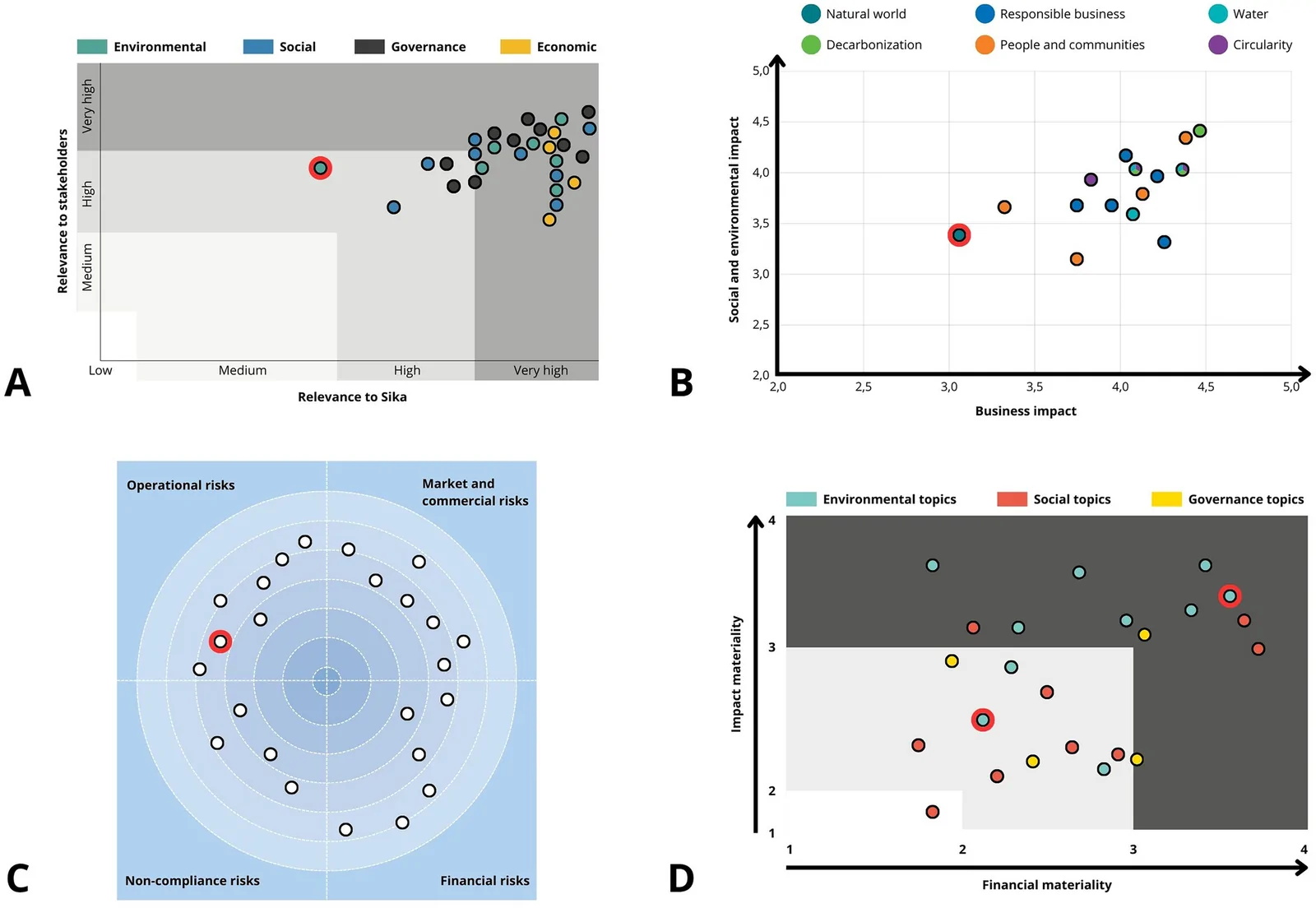
Materiality assessments of leading companies in three priority sectors highlighted by the Taskforce on Nature-related Financial Disclosures (TNFD). These are flagged by TNFD as priority sectors for early adoption of their disclosure framework because of their high dependence on and/or significant impact on nature. It is noteworthy that despite being in a priority sector most companies do not include biodiversity or nature as a material topic, indicating the subjectivity of current materiality assessment. The panels show the materiality matrices of four companies; Panel **A**: Sika AG (Chemicals and Materials), **B**: CRH (Chemicals and Materials), **C**: Boliden (Extractives & Mining), **D**: Unibail-Rodamco-Westfield (Real Estate). Red circles indicate where biodiversity (or environmental impacts) is located on the materiality ranking for each company. Note that Unibail-Rodamco-Westfield split their assessment across operations of current assets and the development of new ones. *Sources*: Sika 2023 sustainability report (p. 84); CRH 2023 sustainability report (p. 16); Boliden Annual and Sustainability Report 2023 (p 53); Unibail-Rodamco-Westfield Universal Registration Document 2023(p 156). For further details see Supplementary Information S1

As already noted, current frameworks and standards for sustainability disclosures let companies determine what social and environmental impacts they deem to be material. This means that companies in a similar sector could independently arrive at quite different assessments of what is a relevant environmental impact to monitor and report on. As an illustrative example, we reviewed the latest materiality assessments of eight leading European companies, across four sectors highlighted by the Taskforce on Nature-related Financial Disclosures (TNFD) as priority sectors because of their high dependence on and/or significant impact on nature. While all except one company present biodiversity as a material topic, it is currently ranked among the lowest in relation to other topics for most of the companies reviewed (Fig. 2, see also SI, Table S1 for more details). This example flags up two noteworthy issues. One is that one of the largest European companies in a priority sector did not even deem biodiversity or nature to be a material topic. Another is that only one company (in real estate) ranked biodiversity as high in relation to development of new property, despite real estate development being a major driver of increasing environmental pressures (Toller et al. 2011; Joof et al. 2024; Fig. 2, Panel D). Interestingly, the same company simultaneously ranked biodiversity as very low in relation to the operation of existing assets.

Critics might argue that companies should not spend resources reporting on what is not material to them but what is financially material changes over time (Antoncic 2019). Climate emissions were not generally considered material before the Taskforce on Climate-related Financial Disclosures (TCFD) developed their guidelines and governments started to enact climate regulations. There is no doubt that we live in an era where environmental impacts are becoming increasingly material (Wang et al. 2021; Almeida et al. 2022; Rana et al. 2022; United Nations Convention to Combat Desertification 2022; Kopp et al. 2023; Ranger et al. 2023; Iglesias et al. 2025). As companies, investors and society become more aware of the relationship between nature, climate and various systemic risks, and their own nature-related dependencies (Almeida et al. 2022; Ranger et al. 2023), the cost of not disclosing and not improving environmental performance will grow under the pressure of court rulings, insurance premiums, the cost of capital and public opinion (Macchi and van Zeben 2021; Worika and Amechi 2023).

### Mandatory disclosure of information that is material to assess impact and systemic risks

While the examples above do not represent a comprehensive analysis, they illustrate the divergence in interpretation that current sustainability disclosure standards allow. In other words, it shows the inconsistencies in what and how environmental impacts are assessed and considered material and thus worthy of reporting across sectors and companies. It suggests that under the emerging regime of sustainability disclosures, information of relevance for understanding biodiversity and nature-related impacts and risks will most likely not be prioritized in reporting for many companies, despite the many such risks already materializing at large scale. It therefore confirms that current disclosures, including the European reporting standards, introduce a subjectivity into the assessment of impact that risks fueling misinformation, reduce credibility and comparability between companies and sectors, and ultimately undermine the capacity of public actors to assess aggregate and cumulative impacts, and thus set reliable environmental targets. It also reduces the ability of investors to assess systemic risks. In contrast, an approach that determines and makes mandatory a specified set of non-financial disclosures based on what environmental pressures and impacts science indicates are most relevant to a specific sector, would address these issues. Such proposals exist (Wassénius et al. 2024), and would contribute to substantially reduce the burden of sustainability reporting relative to the current system, while providing the information necessary to begin to mitigate systemic risks (Crona et al. 2021).

While mandatory reporting across all segments of our value chains, including SMEs, is the only way to enable reliable scope 3 analysis of any environmental impacts, we recognize that current regulatory environments and political discourse in some regions make this an unfeasible short-term goal. Voluntary disclosures of direct operations are therefore a first and important step toward achievement of an, ultimately, improved corporate data generation system to support systemic risks assessments and truly sustainable investments. Voluntary disclosures can also enhance a company’s license to operate by demonstrating transparency and supporting public agencies and society as end users of sustainability data.

### Transparent and accessible reporting that benefits multiple stakeholders

A reformed approach to corporate environmental sustainability disclosures, as described above, is paramount if we want companies to disclose information that can help assess the mounting threat of systemic risks, and not just short-term risks to their own operations and investments. However, the information will only be useful to decision-makers and end users (ranging from public agencies to investors and the academic community) if it is accessible. An improved agenda for corporate sustainability reporting will therefore rely on the simultaneous creation of a standardized public data repository where the reported information can be accessed in a transparent, consistent, and reliable format.

**Fig. 3:**
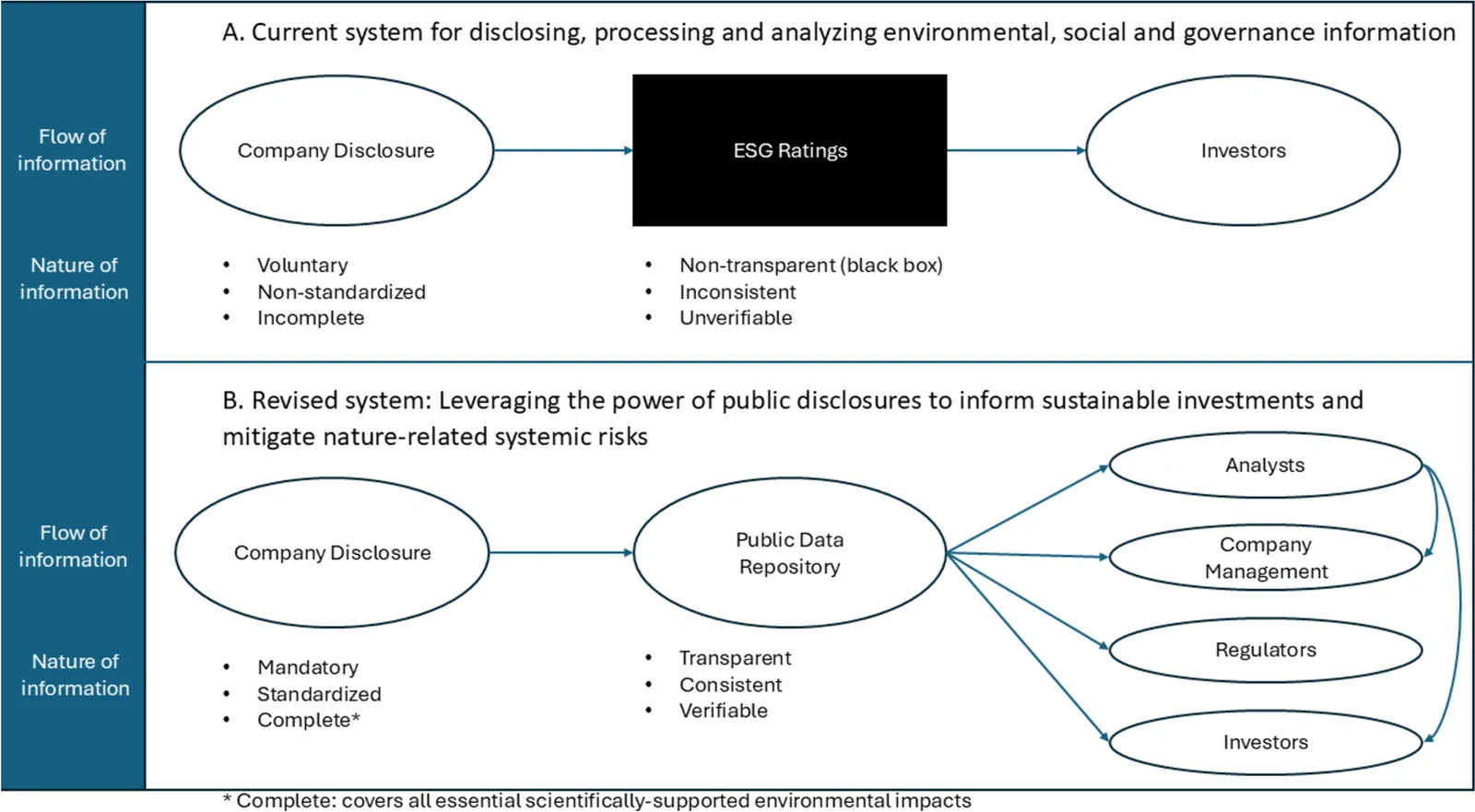
Replacing the black box of ESG—Contrasting status quo with a revised system for corporate environmental disclosures. In the current system, companies voluntarily disclosure non-financial environmental data, which in many jurisdictions is non-standardized and incomplete since disclosures are premised on a financial materiality assessment. Based on these disclosures, ESG rating services use proprietary (black box) methods leading to non-transparent, inconsistent, unverifiable results. In the proposed revised system, companies would be required to report on scientifically based essential environmental impacts to a public data repository resulting in transparent, consistent and reliable data. The role for analysts would shift to providing decision-relevant information, much like the current role for financial analysts

This repository could be used for a variety of purposes including informing company management, regulators, investors, and academic analysis (Blum and Mathon 2023). But importantly it could spur a new guild of analysts focused on making environmental disclosures actionable and easily accessible to financial actors, providing tools to help investors make sense and analyze the large amounts of publicly available environmental disclosures and integrating this information with financial risk measures (Fig. 3). This would emulate analytical services that have provided decision-relevant information based on corporate financial data ever since financial disclosures became standardized, but would constitute a radical novelty and significant improvement in analytical quality and potential for science-informed sustainable investments.

A revised system for storing disclosed information has already been proposed by several prominent organizations (Autorité des Marchés Financiers and Autoriteit Financiële Markten 2020; International Organization of Securities Commissions 2021). Such a system would remove the need to create proprietary products based on voluntary, non-standardized, and incomplete information, which then generates non-transparent and unreliable results that are inconsistent across products, as is the case with current ESG ratings (Escrig-Olmedo et al. 2019; Dimson et al. 2020; El-Hage 2021; Berg et al. 2022) (Fig. 3). It would further remove the current intrinsic information asymmetry, where companies have the upper hand, and where a lack of access to information and common reference points among investors prevent those who share a common sustainability ambition from acting in concert when they engage with companies or make decisions about inclusions and exclusions of portfolios (Altendorfer et al. 2025). Achieving the reduced information asymmetry will require a wide (ultimately mandatory) input into such a data repository, and input could be the responsibility of companies, regulators or even accounting firms at the request of their clients. A governance model of the digital commons in line with, e.g., the Global Legal Entity Identifier Foundation (GLEIF) or the World Intellectual Property Organization (WIPO) would ensure its credibility. We recognize that actors specializing in developing proprietary ESG data based on current voluntary and incomplete disclosures may resist proposals for a public data repository (Blum and Mathon 2023). However, new business opportunities providing value-added analysis and data-driven solutions will ensue for such actors.

## Conclusion

The current geopolitical turbulence and the concomitant flux of corporate sustainability reporting is certainly worrying but can also be seen as a golden opportunity for sustainability science and scientists to provide much needed guidance that can simultaneously help reduce, refine, and improve sustainability reporting by identifying the most prioritized mandatory environmental disclosures and support the development of an open-access disclosure repository. Disclosures that capture the most environmentally material impacts of a given company or sector would radically improve the ability of investors, regulators, and public agencies to appraise environmental pressures, improve corporate accountability, and mitigate growing systemic risks. It would therefore likely also increase or maintain the social license for companies to operate (Morrison 2014).

To address the parallel needs of investors and society, environmental data should be viewed in the same way as financial data, with scientifically informed and standardized disclosures that are made available on a public platform (Blum and Mathon 2023). Like financial data, sustainability data aim at reducing information asymmetry between stakeholders. This is really the only way to also ensure that the CSRD and similar reporting initiatives remain effective in driving transparency on sustainability impacts without unduly burdening companies—in line with aims of the European Commission and the TNFD. Getting to such a system will require more governments to make socio-environmental impact disclosures mandatory and also enact and enforce environmental regulations.

A revised system would improve the capacity of companies and investors to more accurately assess both direct environmental risks and indirect systemic risks. It would enable financial institutions to contribute to a sustainability transformation, and ESG funds to invest in truly sustainable activities, not just the firms that are good at reducing their own immediate risks (be they physical, reputational or regulatory). It would further significantly increase the capacity of public agencies and academics to develop reliable estimates of aggregate and cumulative human pressures on the planet, which in turn increases our collective ability to set accurate and meaningful environmental targets.

Proceeding with status quo is akin to flying in an airplane without navigation systems. Just as one would not fly on such an unsafe airplane, society should not continue without knowledge of the nature and magnitude of corporate impacts and the risks that they impose on us all.

## Supplementary Information

Below is the link to the electronic supplementary material.

Supplementary file1 (PDF 198 KB)
