# Supplementary material for: A golden opportunity: Corporate sustainability reporting as a key lever to address nature-related risks
Source: Ambio. 2026 Mar 14;55(10):2321–31. doi: 10.1007/s13280-026-02376-0 (PMC13554042; doi:10.1007/s13280-026-02376-0)
Supplement: Supplementary file 1 [file 13280_2026_2376_MOESM1_ESM.pdf]

Supplementary Information

Ambio

A golden opportunity: Corporate sustainability reporting as a key lever to address nature-related risks

Betrice Crona[1,2*], Stephen Polasky[3], Giorgio Parlato[1], Véronique Blum[4], Maxime Mathon[5]

[1] Stockholm Resilience Centre, Stockholm University, Stockholm, Sweden.
[2] Global Economic Dynamics and the Biosphere, Royal Swedish Academy of Science, Stockholm, Sweden.
[3] Dept. of Applied Economics, University of Minnesota, St Paul, Minnesota, US.
[4] Centre de Préparation à l'Expertise Comptable, Université Grenoble des Alpes, Grenoble, France
[5] Ascend, Paris, France.

*Corresponding author: Email: Beatrice.Crona@su.se

Contents

Methods for assessing material topics identified by leading companies in four priority sectors in Europe ... 3

Results of sustainability report review ... 4

Methods for assessing material topics identified by leading companies in four priority sectors in Europe

We selected four sectors (Infrastructure & Real Estate, Extractives & Mining, Chemicals & Materials, and Utilities & Power), all of which are highlighted by the Taskforce on Nature-related Financial Disclosures (TNFD) as priority sectors (for early adoption of their disclosure framework) because of their high dependence on and/or significant impact on nature.

Within each sector we selected two large-cap companies from across Europe and reviewed their most recently published sustainability reports to provide an illustrative example of how some of the leading European companies in the aforementioned priority sectors currently assess the materiality of biodiversity.

In January of 2025 we downloaded the most recently published sustainability reports available for the selected companies and reviewed these to assess i) if (and how) biodiversity was ranked in a materiality assessment, ii) if a double materiality assessment was done, iii) how the topic of biodiversity was dealt with broadly. That is, if there was any other information that could indicate how the company viewed the materiality of biodiversity to the business. Note that a “double materiality” assessment refers to a process of identifying business material topics using a broadened concept of materiality, to not just focus on financially material topics but also include potential impacts on stakeholders and society and is recently mandated for companies reporting under the Corporate Sustainability Reporting Directive.

Sources: Sika 2023 sustainability report; CRH 2023 sustainability report; Boliden Annual and Sustainability Report 2023; Unibail-Rodamco-Westfield Universal Registration Document 2023, Anglo American plc Sustainability Report 2023; Centrica Strategic report/Annual Report and Accounts 2023; E.ON Integrated Annual Report 2023; Vonovia SE Combined Management report 2023.

Results of sustainability report review

Below we summarize the findings, which form the basis for statements made in the main article. All companies reviewed had conducted some form of double materiality assessment, where non-financial, stakeholder-oriented concerns we also mapped. The companies vary in their disclosure of the materiality assessment process, and in how they present the results and their material topics. Some merely highlighted and discussed material topics, while others provide a systematic overview of their materiality assessment where it is clear how the materiality of different topics rank in relation to each other. In summary, while biodiversity features as a material topic for all companies, except one (Centrica), it is currently ranked among the lowest in relation to other topics for most of the companies reviewed.

Table SI1. Treatment of biodiversity (and nature) in the sustainability reports of two leading European companies in each of four priority sectors (as identified by the Taskforce on Nature-related Financial Disclosures (TNFD)).

**INFRASTRUCTURE & REAL ESTATE**
**Vonovia SE** (HQ: Germany): Europe's largest residential real estate company, which not only owns and manages a vast portfolio of apartments but also develops new housing projects and modernizes existing stock.<br>*Biodiversity is rated as being of high (but not very high) business relevance in their materiality assessment, yet they point out that "Vonovia's economic activities do not significantly harm the achievement of EU environmental objective 6 (protection and restoration of biodiversity and ecosystems), as Vonovia only builds in designated areas and with a building permit" (p 55 of sustainability report). They do not make a distinction between biodiversity impacts stemming from current operations of real estate assets versus new developments.*<br><br>**Unibail-Rodamco-Westfield** (HQ: France): A major commercial real estate developer and operator, known for its shopping centres, offices, and mixed-use projects across Europe and the US, where it develops new complexes and manages them post-completion.<br>*URW have conducted a double materiality assessment in 2023 where they identified a total of 23 topics, of which 12 were "identified as material for URW with regard to their level of importance, from a financial and impact perspective" (p 156, Sustainability Report). URW divides biodiversity-related impacts into those stemming from operation of their current stock of real estate, and those resulting from new development projects. The latter is considered one of the 12 highly material topics for the company (see materiality matrix, p 156).*
**EXTRACTIVES & MINING**
**Anglo American plc** (HQ: UK): A global mining corporation extracting metals and minerals (e.g., diamonds, copper, platinum) in various regions worldwide.

In 2023 Anglo-American conducted a double materiality assessment, resulting in a materiality matrix where biodiversity was ranked as being of medium importance in terms of both environmental and financial (business) impact (p 23). The company outlines their approach to biodiversity which consists primarily of mine rehabilitation (p 62). It mentions drivers of biodiversity loss, such as water use and introduction of invasive species that can have impacts on what they call Significant Biodiversity Features. Identification of SBF is part of their internal Biodiversity Standards, but this was not assessed in this analysis.

Boliden AB (HQ: Sweden): A metals and mining firm engaged in the exploration, extraction, and refining of base and precious metals in Northern Europe.

In 2023 Boliden AB conducted a double materiality assessment to define their scope for reporting (p 72). In the 2023 sustainability report they do not present a conventional materiality matrix, but rather a risk mapping, where climate change and other environmental impacts fall under operational risks and are ranked amongst the lower of operational risks, but also in relation to market and commercial risks, non-compliance risks, and financial risks (p 53). The report mentions an ambition to contribute to increased biodiversity in all regions where Boliden operates, starting with avoiding and minimizing and restoring their impact where possible and compensating for any residual impact. They have biodiversity management plan for certain sites.

CHEMICALS & MATERIALS

Sika AG (HQ: Switzerland): Aa Swiss multinational specializing in chemical products for the building sector, including concrete admixtures, waterproofing, and sealing systems.

Sika AG conducted a materiality analysis in 2021/2022 (approved in 2022). In their 'Material topic boundaries' document, biodiversity and nature is included as a material topic in relation to a number of sub-topics which structure the corresponding section in the sustainability report (p 101). They present a materiality matrix (p 48), where biodiversity ranks among the lowest of all business-material topics. Sika AG discuss their process of gathering more information about nature-related issues and their analysis of company dependencies and impacts using the LEAP framework of the TNFD, and tools such as ENCORE and IBAT)(p 101). There is no biodiversity management plan.

CRH plc (HQ: Ireland): A multinational building materials company operating across various segments of the construction sector, including cement, aggregates, and building products.

CHR present the results of a double materiality assessment in their 2023 report. Biodiversity is a material topic but, along with air quality, ranks the lowest in perceived importance for both business and social/environmental impact (p 16, see also Figure 2 in main text). CHR has a section devoted to discussing biodiversity in relation to their operations and elaborate on how they currently engage to assess and reduce their biodiversity impacts, and ambitions for promoting it in their operations, including a biodiversity management plan (p 41).

UTILITIES & POWER

Centrica plc (HQ: UK) A leading energy and services company, providing electricity and gas to residential and business customers through brands like British Gas, and offering energy solutions and services globally.

Their sustainability report does not mention biodiversity, nature, or any nature-related risks. Under 'our foundations' they state that "Monitoring and managing our wider environmental impact is crucial" (p33). However, Centrica reports only on greenhouse gas emissions and on water use, waste generated and instances of non-compliance with environmental authorisation. Despite known environmental effects of natural gas production (one

of their cornerstones of Centrica's energy supply) that go beyond GHG emissions, water use and waste, they do not discuss any environmental impacts or provide a systematic materiality assessment in their sustainability report.

E.ON SE (HQ: Germany): A major European energy company focusing on electricity generation and distribution, renewable energy projects, and providing energy solutions and services to consumers and businesses across Europe.

In 2022 they undertook an analysis of the extent to which its business model impacts biodiversity and divided the analysis into their own dependencies on biodiversity, and their impacts on biodiversity. They found that the highest ecosystem service dependence was hydroelectricity, with flood and storm protection as key services, and that their main impacts stem primarily from energy production from biomass, hydropower, and heat plants (p48). Biodiversity is also discussed in relation to impacts of E.ON's biomass-fired assets (p83). However, when E.ON conducted a materiality assessment in 2023, in accordance with the Non-Financial Reporting Directive, and applying the double materiality principle taking CRSD requirements into account, this resulted in three material issues climate-change mitigation, energy affordability and reliable energy supply as the most material issues (p30). They roved no systematic materiality assessment in their sustainability report.
